# Association between a dietary pattern high in saturated fatty acids, dietary energy density, and sodium with coronary heart disease

**DOI:** 10.1038/s41598-022-17388-5

**Published:** 2022-07-29

**Authors:** Nur Ain Fatinah Abu Bakar, Aryati Ahmad, Wan Zulaika Wan Musa, Mohd Razif Shahril, Nadiah Wan-Arfah, Hazreen Abdul Majid, Carmen Piernas, Ahmad Wazi Ramli, Nyi Nyi Naing

**Affiliations:** 1 Faculty of Health Sciences, Universiti Sultan Zainal Abidin (UniSZA), Kuala Nerus, Terengganu, Malaysia; 2 Nuffield Department of Primary Care Health Sciences, University of Oxford, Oxford, UK; 3 Centre for Healthy Ageing and Wellness (HCARE), Faculty of Health Sciences, Universiti Kebangsaan Malaysia, Kuala Lumpur, Malaysia; 4 Department of Social and Preventive Medicine, Faculty of Medicine, Universiti Malaya, Kuala Lumpur, Malaysia; 5 Medical Department, Hospital Sultanah Nur Zahirah (HSNZ), Kuala Terengganu, Terengganu, Malaysia; 6 Faculty of Medicine, Universiti Sultan Zainal Abidin (UniSZA), Kuala Terengganu, Terengganu, Malaysia

**Keywords:** Cardiovascular diseases, Cardiology, Nutrition

## Abstract

This study aimed to determine the association between dietary pattern (DP) and coronary heart disease (CHD) among high-risk adults as determined by metabolic syndrome (MetS) criteria in Malaysia. This cross-sectional study involved 365 participants with (CHD = 178; non-CHD = 187) who were recruited from selected health clinics. Dietary intake was measured using a 189-item semi-quantitative foods frequency questionnaire (FFQ) whilst anthropometry and clinical data were measured by trained researcher and biochemical data were obtained from medical records. The reduced rank regression (RRR) method was used to derive DPs scores and binary logistic regression was used to assess the associations between identified DPs and CHD. The main DP found in this study was characterised by “high saturated fatty acid (SFA), high dietary energy density (DED), high sodium”. This DP, which is attributed to high consumption of coconut-based dishes, fast foods and snacks, rice dishes, fat spread, seasoning sauces, salted and processed foods, and low intake of fruits, green leafy vegetables, white rice and other vegetables were associated with CHD (OR:1.32, 95% CI:1.03, 1.69) *p* value = 0.026 when, adjusted for age, sex, race, education level, household income, family history of CHD, marital status, smoking status, physical activity, stress level and BMI. This study suggests that individuals with a DP of high SFA, high DED, and high sodium have a significantly increased likelihood of having CHD compared to those who do not practice this DP.

## Introduction

Cardiovascular diseases (CVDs) including coronary heart disease (CHD), cerebrovascular disease or accident (CVA), peripheral arterial disease, rheumatic disease are recognised as the number one cause of death globally^1^. Rising trends in the prevalence and fatalities of CVD, particularly CHD have heightened global attention to the prevention and control of these diseases^2,3^. In Malaysia, CHD was the leading cause of death in 2019, accounting for 15% of all fatalities that requiring immediate attention^4^.

Modifications of lifestyle which involve weight reduction, smoking cessation and adherence to healthy dietary patterns are important to ameliorate CHD and other CVDs risk factors^5^. Dietary intake, in particular, has longstanding contributions towards CHD development. The impacts of single nutrients intake such as intakes of dietary fat, sugar, carbohydrate, fibre, etc. with CHD risks have been largely addressed by researchers in epidemiological studies over the past decades^6^.

Recently, there is growing evidence suggesting the importance of dietary pattern (DP) instead of single nutrients with regards to the non-communicable diseases (NCDs) and CVD risk. According to a recent study, a healthy DP which is characterized by high intake of soy, vegetables, fruits, tea, tomato products, bread, fish, margarine and dairy, and low in rice, red meat, coffee, alcohol, sugar-sweetened beverages, and eggs was proven to be associated with a lower risk of diabetes mellitus (DM) and CHD risk^7^. In contrast, a latest published prospective cohort study among UK Biobank participants had proven that a DP high in chocolate confectionery, butter, refined bread, and table sugar and preserves, together with low intakes of fresh fruit, vegetables, and wholegrain foods, are associated with an increased risk of CVD and all-cause mortality^8^. Several research on DP have been conducted in Malaysia involving individuals at risk of metabolic syndrome (MetS)^9–11^; however, no study has yet reported on the specific kind of DP linked to CHD in this high-risk population, and majority of studies utilised principal component analysis (PCA) in DPs derivation rather than reduced rank regression (RRR) analysis.

The RRR analysis has been proposed as a new dimension-reduction technique in the identification of DPs, and it is more flexible and powerful than the classic PCA^12^. Overall assessment of DPs with the combination of food groups is a better approach than using a single nutrient in evaluating the role of diet in the development of chronic diseases^13^. Evidences about DPs within certain disease populations are useful to propose a new perspective of CVD guideline based on DP. Moreover, dietary advices that focus on foods rather than individual nutrient could help avoid confusion, as DPs are readily translatable to dietary practice because foods are eaten in food matrix instead of nutrients^14^. The aim of this study was to determine the association between DP and CHD among high-risk adults as determined by MetS criteria in Malaysia.

## Methods

### Study design

This cross-sectional study involved 365 participants, comprised of 178 CHD and 187 non-CHD participants with the risk of MetS in Malaysia. This study was conducted from February 2018 until September 2020. The selections of medical clinics were based on purposive sampling from every region of Malaysia; east coast, central, south, north, and east of Malaysia. From every clinic, consecutive sampling was applied for the selection of participants to obtain the pre-determined sample size. The sample was drawn from the list of referral patients in the clinics and subjects that meet the eligibility criteria were included in this study. The sample size, 368, was determined by using two independent means for the numerical independent variable (age) in Power and Sample Size Calculation (PS) software^15^ with 20% dropout rate. The calculated sample size was considered adequate based on the most prevalent independent risk factor of CHD e.g. age.

### Study participants

The inclusion criteria of this study were adults aged 30 to 64 years old, from both genders and have at least three or more of the metabolic syndrome risk factors (elevated waist circumference (men: > 90 cm; women: > 80 cm), elevated triglyceride (TG): ≥ 1.7 mmol/l or on drug treatment for elevated TG, reduced HDL-cholesterol (men: < 1.0 mmol/L; women: < 1.3) or on drug treatment for reduced HDL-C, raised blood pressure (systolic: ≥ 130 mmHg or diastolic: ≥ 85 mmHg) or on antihypertensive drug treatment, elevated fasting plasma glucose: ≥ 5.6 mmol/l) or on drug treatment of elevated glucose). The metabolic syndrome criteria used in this study were based on a harmonized joint interim statement for the definition of metabolic syndrome issued by established organisations^16^. Metabolic syndrome criteria were used to avoid significant difference between groups thus reducing risk of bias. For CHD group, only those who had been diagnosed within the previous two years of recruitment were included in this study. Exclusion criteria for this study were unstable patients (e.g., patient admitted to the ward, unconscious patients), pregnancy, patients with cognitive impairment (e.g., senile, dementia), mute and deaf patients.

All potential research participants understood the procedure, risks, and benefits of the study. They were also informed that they could decide to withdraw from the study at any time without providing reasons. All respondents provided their informed consent before answering the questionnaires including the consent for open access publication. Each method was performed in accordance with the relevant guidelines and regulations by the Declaration of Helsinki. This study received ethical clearance from the National Medical Research Register from the Malaysian Ministry of Health (NMRR No-18-269-39671) and UniSZA Human Research Ethics Committee (UniSZA. C/2/UHREC/628-2(30)).

### Variable and data collection

Participants who agreed to participate in this study were interviewed for socio-demographic data, lifestyle and behavioral factors (e.g. physical activity, stress level, smoking status), medical history, and dietary intake using a set of questionnaires. For anthropometry data, weight (kg), height (cm) and waist circumference (cm) were measured by trained researchers using Tanita digital weighing scale, a stadiometer and a SECA measuring tape, respectively. BMI was derived using the metric system (kg/m^2^)^17^, and was classifed into underweight, normal, overweight or obese based on the standard BMI cut-off points^18^. Central adiposity, which assessed using WC, was classified according to cut off point of > 90 cm for men and > 80 cm for women^19^. Meanwhile, systolic and diastolic blood pressure measurement were measured using an automated Omron blood pressure (HEM7203) by a health professional. Biochemistry results (e.g. fasting serum lipid and blood glucose profile) were retrieved from medical records.

Semi-quantitative food frequency questionnaire (FFQ) was used to capture the habitual dietary intake of the participants over the previous year prior to CHD diagnosis. The used FFQ was modified from the Malaysia Adult Nutrition Study (MANS)^20^ and was validated against a weighed food record^21^. The FFQ consists of 189 food items and a selection of options related to the frequency of consumption for each food listed (e.g. times per day, daily, weekly, monthly). Participants were required to estimate how often on average they had taken the food items with the aid of the local household measurement photographs. The estimation of daily energy, macronutrient and micronutrient intakes based on the amount of food taken by participants daily were calculated using a database which was developed based on the Nutrient Composition of Malaysian Food^22^, United States Department of Agriculture (USDA) nutrient database^23^, and the Singapore Food Database^24^.

Physical activity level of the subject was assessed using the Malay International Physical Activity Questionnaire Short Form (IPAQ), which have been proven its reliability and validity in 12 different countries^25^. The Malay IPAQ short version used in this study, was previously pilot-tested and pre-validated in previous 2011 NHMS^26^. Meanwhile, the stress level of each participant was assessed using the Malay version of the 10-item Perceived Stress Scale (PSS-10)^27^, which previously tested with results of satisfactory level of validity and reliability to assess stress perception^28^.

### Data analysis

#### Statistical analysis

All data were analysed using IBM SPSS Statistics 27 (Armonk, NY: IBM Corp) yet the RRR were run using SAS software version 9.4 (SAS Institute, Cary NC) for derivation of the DP. The Kolmogorov–Smirnov test, was used to assess normality for the continuous variables for this study population. The test revealed that the majority of continuous data were non-normally distributed; however, parametric test was used in this study since the central limit theorem states that violations of the normality assumption should not cause major problems if the sample size is large enough (> 30 or 40), and parametric tests can be used even if the data are not normally distributed^29–31^. Descriptive data relating to demographic, socio-economic status, behavioral risk factors, medical conditions, and dietary intake of total study participants were compared against tertiles (T1–T3) of dietary patterns using the chi-square tests for categorical variables and One-way ANOVA for continuous variables.

Simple logistic regression was used to determine the correlation between CHD (dependent variable) and independent variables (e.g., demographic, socioeconomic status, behavioral risk factors, medical conditions, and dietary intake). Binary logistic regression was used to determine association between identified DPs (total factor loading for a whole DP, and between DP tertiles (T2 vs. T1; T3 vs. T1) and CHD. The independent risk factors associated with CHD risk were identified and controlled for its potential confounding effect in the binary logistic regression analysis. A variable is considered as potential confounding variables, if (1) it is associated with the exposure (independent variable) either causally or not, (2) it is associated with the outcome (dependent variable), and (3) it must not involve in the causal pathway between the exposure and outcome^32^.

Considering these rules, three regression models were generated by adding variables to the previous model at each step, by which model 1 was adjusted for age and sex, model 2 was adjusted with additional of other socio-demographic factors (race, education level, income, family history of CHD, marital status) and model 3 was adjusted with additional of environmental factors (smoking, physical activity, stress level and BMI) to model 2. Independent variables like waist circumference, biochemical profile (e.g. lipid profile, blood glucose profile) and clinical data (e.g. blood pressure) were not adjusted since these parameters involved in the causal pathway between exposure and outcome (CHD). Furthermore, all our study participants were recruited with the presence of the abnormality of these parameters (part of MetS criteria). Multicollinearity was tested using tolerance and the variance inflation factor (VIF).

Numerical variables are presented in mean and standard deviation (SD), whilst categorical variables are presented in frequency (n) and percentage (%). For all assessed parameters, statistical significance was set at *p* value < 0.05.

#### Reduced rank regression analysis (RRR)

The RRR analysis was applied to derive DPs using SAS software version 9.4 (SAS Institute, Cary NC). This method was used to determine the linear function of predictor variables (food groups) by maximizing the explained variation in responses (disease-related nutrients)^12^. Identification of DPs involved several important steps: i. Simple logistic regression analysis between single nutrient intake and CHD (Supplementary Table S1), ii. Derivation of interested DPs by including nutrients which showed significant univariate associations as response variables in the RRR, iii. A priori selection of DPs with explained variation > 20%^8^.

In this study, the 189 food items were categorized into 30 food groups (g/day) that were classified based on their nutritional profiles. These food groups were then used as predictor variables in RRR analysis to determine their linear combinations that elucidated as much as possible variation in the response variables (e.g., nutrients: dietary energy density (DED), dietary fat intake, etc.). Three combinations of variables: DED (kcal/g), SFA (g/1000 kcal), sodium (mg/1000 kcal) used in this study were chosen based on their established relationship with CHD from the previous literatures^33–35^.

Every nutrient was converted into the standard conversion unit in which DED was calculated by dividing total energy intake (kcal) with total food weight (g) by excluding beverages^36^. In order to report adjusted intake for energy consumption, SFA (g/day) and sodium (mg/day) intake values were determined by respectively dividing them with total daily energy intake (kcal) and then multiplied by 1000 kcal^37,38^. For the calculation of the DP score, all predictor variables (30 food groups) and response variables were set in SAS software using specific coding for RRR analysis. The RRR model calculates DPs z-scores for each participant as a linear, weighted combination of all of their standardized food group intakes by using weights unique to each dietary pattern.

Each participant obtained an individual z-score for each DP derived; a higher z-score corresponded to higher adherence to the identified DP. Intake of foods with a positive factor loading increased the DP z-score, whilst intake of foods with a negative factor loading decreased the DPs z-score. Food groups with factor loading ≥ 0.20 and ≤ − 0.20 were significant and considered as the largest positive and negative contribution to the dietary pattern z-scores, respectively^39^. Participants were categorised into tertiles (T1–T3) based on factor score of the identified DP, where tertile 1 (T1) represented the lowest adherence while tertile 3 (T3) was the highest adherence to that pattern.

## Results

A total of 365 participants (178 CHD (48.8%) 187 non-CHD (51.2%)) with mean age 51.6 ± 9.1 years old were included in this study. Overall, the study participants have high proportion of men, Malay, married, low household income (B40 (B1) category), low education level (secondary school), non-smokers, low level of physical activity, and overweight (Tables 1, 2).

**Table 1:** General characteristics of participants (demographic, socioeconomic, and behavioural risk factors in main dietary patterns based on tertiles (N = 365).

General characteristics	Total (N = 365)	Dietary pattern 1 (High DED, high SFA, high sodium)			
		Tertile 1 (N = 121)	Tertile 2 (N = 122)	Tertile 3 (N = 122)	*p* value*
Total CHD cases (%)	48.8	48.8	43.4	54.1	0.250^a^
Total non-CHD cases (%)	51.2	51.2	56.6	45.9	
**Demographics**					
Sex (%)					0.204^a^
Women	46.3	52.9	42.6	43.4	
Men	53.7	47.1	57.4	56.6	
Age(year)	51.6 ± 9.1	53.1 ± 7.9	50.4 ± 10.3	51.2 ± 8.9	0.062^b^
Ethnicity (%)					0.570^a^
Malay	81.6	78.5	86.1	80.3	
Chinese	6.6	6.6	5.7	7.4	
Indian	9	6.6	12.3	9	
Others (e.g*. Kadazan Dusun, Bajau,* *Bidayuh*)	2.8	8.3	0	0	
Family history of CHD (%)					0.150^a^
No	66	60.3	72.1	65.6	
Yes	34	39.7	27.9	34.4	
Marital status (%)					0.012^a^
Married	75.6	76	83.6	67.2	
Single	24.4	24	16.4	32.8	
**Socioeconomic status**					
Household Income (%)					0.848^a^
B40 (B1) < RM2500	51.8	52.1	48.4	54.9	
B40 (B2) RM2501-3170	12.3	12.4	11.5	13.1	
B40 (B3) RM3171-3970	13.4	13.2	12.3	14.8	
B40 (B4) RM3971-4850	9.0	8.3	12.3	6.6	
M40 RM4851-10970	12.9	14.0	14.8	9.8	
T20 > RM10971	0.5	0	0.8	0.8	
Education group (%)					
Higher degree (college or university degree, or professional qualifications)	15.9	12.4	18.0	17.2	0.483^a^
*Diploma*/*STPM*/Vocational qualifications	22.2	22.3	25.4	18.9	
PMR*/SPM* (secondary school)	61.9	65.3	56.6	63.9	
**Behavioural risk factors**					
Smoking status (%)					0.739^a^
Non-smoker	72.9	71.9	71.3	75.4	
Smoker	27.1	28.1	28.7	24.6	
Physical activity (%)					0.114^a^
Low	43.6	50.4	45.9	34.4	
Moderate	38.4	33.1	35.2	46.7	
High	18.1	16.5	18.9	18.9	
Perceived stress score, Mean ± SD	13.5 ± 5.8	12.4 ± 6.3	14.0 ± 5.6	14.0 ± 5.2	0.042^b^

^a^Pearson Chi-square test.

^b^One-Way ANOVA. *Significant level at *p* value < 0.05. *B40* Bottom 40%, *CHD* Coronary heart disease, *DED* Dietary energy density, *M40* Middle 40%, *PMR* *Penilaian Menengah Rendah*, *SD* Standard deviation, *SFA* Saturated fatty acid, *SPM* *Sijil Pelajaran Malaysia*, *STPM* *Sijil Tinggi Pelajaran Malaysia*, *RM* *Ringgit Malaysia*, *T20* Top 20%.

**Table 2:** General characteristics of participants (anthropometry, biochemical, and clinical data) in main dietary patterns based on tertiles (N = 365).

General characteristics	Total (N = 365)	Dietary pattern 1 (High DED, high SFA, high sodium)			
		Tertile 1 (N = 121)	Tertile 2 (N = 122)	Tertile 3 (N = 122)	*p* value*
**BMI classification (%)**					0.055^a^
Underweight < 18.5 kg/m^2^	0.3	0	0	0.8	
Normal 18.5–24.9 kg/m^2^	31.0	27.3	36.1	29.5	
Overweight 25–29.9 kg/m^2^	37.3	33.9	31.1	46.7	
Obese I 30–34.9 kg/m^2^	17.5	19.0	20.5	13.1	
Obese II 35–39.9 kg/m^2^	9.9	12.4	8.2	9.0	
Obese III > 40 kg/m^2^	4.1	7.4	4.1	0.8	
Body mass index, Mean ± SD, kg/m^2^	28.4 ± 5.9	29.6 ± 6.9	28.0 ± 5.9	27.6 ± 4.7	0.017^b^
Waist circumference Mean ± SD, cm	92.7 ± 12.0	94.6 ± 13.3	91.2 ± 10.7	92.4 ± 11.8	0.081^b^
**Blood lipid profile, Mean ± SD, mmol/L** **(n = 354)**					
Total cholesterol	5.3 ± 1.2	5.3 ± 1.3	5.2 ± 1.2	5.2 ± 1.1	0.735^b^
Triglyceride	1.6 ± 0.5	1.7 ± 0.5	1.5 ± 0.5	1.7 ± 0.5	0.012^b^
HDL cholesterol	1.1 ± 0.2	1.1 ± 0.2	1.1 ± 0.2	1.1 ± 0.3	0.094^b^
LDL cholesterol	3.0 ± 1.0	3.0 ± 1.0	3.1 ± 1.1	2.9 ± 0.9	0.282^b^
**Blood sugar profile, Mean ± SD**					
Fasting blood sugar (FBS) mmol/L (n = 354)	6.9 ± 1.7	7.0 ± 1.6	6.9 ± 1.8	6.8 ± 1.7	0.462^b^
HbA1c (%) (n = 349)	7.0 ± 1.5	7.2 ± 1.6	7.0 ± 1.4	6.8 ± 1.3	0.106^b^
**Blood pressure (mm/Hg), Mean ± SD**					
Systolic blood pressure	137.0 ± 14.2	137.0 ± 14.3	137.5 ± 13.8	136.4 ± 14.7	0.816^b^
Diastolic blood pressure	82.6 ± 8.8	82.9 ± 9.0	82.9 ± 8.8	81.9 ± 8.5	0.583^b^

^a^Pearson Chi-square test.

^b^One-Way ANOVA. *Significant level at *p* value < 0.05. *DED* Dietary energy density, *FBS*: Fasting blood sugar, *HDL*: High density lipoprotein, *LDL*: Low density lipoprotein, *SD* Standard deviation.

**Figure 1:**
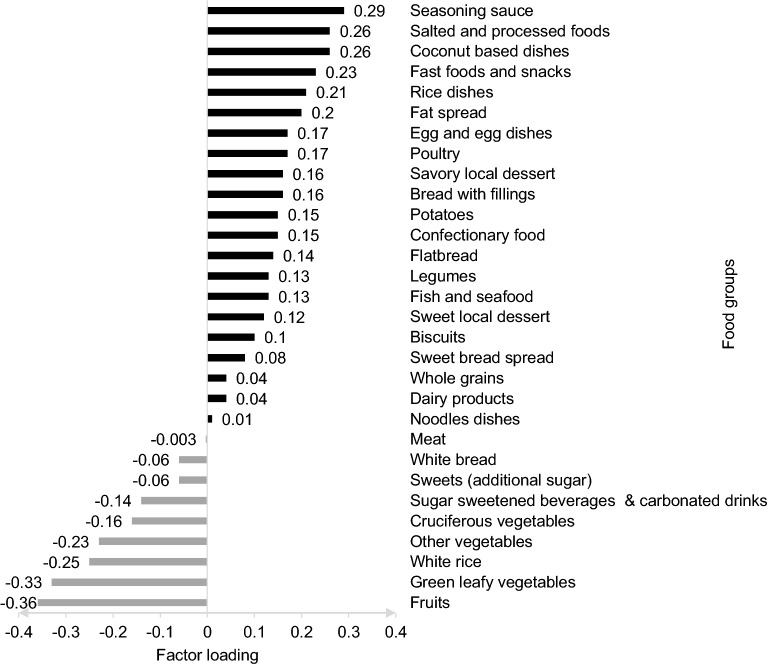
Factors loadings for DP1 “High SFA, high DED, high sodium”. DP1 is attributed by food groups with factor loadings of  ≥ 0.20 and ≤ −0.2.

The RRR analysis identified one major DPs (DP1) that explained 22% of variation in the response variables (SFA, DED, sodium). The DP1, which was named as “high SFA, high DED and high sodium”, had moderate to strong correlation for sodium density (r = 0.313), DED (r = 0.530) and SFA (r = 0.788) (Table 3). This DP1 was characterized by positive factor loadings for coconut-based dishes, fast foods and snacks, rice dishes, fat spread, seasoning sauce, salted and processed foods, and negative loadings for fruits, green leafy vegetables, white rice and other vegetables. Factor loadings for DP1 “high SFA, high DED, high sodium” are presented in Fig. 1.

**Table 3:** Explained variation (%) in food intakes and response variables for each dietary pattern as assessed using reduced rank regression (RRR) and correlation coefficient between dietary patterns and response variables (n = 365).

Dietary patterns	Explained variation (%)					Correlation coefficient		
	Food intakes (current)	Response (current)	SFA (g/1000 kcal)	DED (kcal/g)	Sodium (mg/1000 kcal)	SFA (g/1000 kcal)	DED (kcal/g)	Sodium (mg/1000 kcal)
DP1	5.49	21.52	40.10	18.11	6.34	0.788	0.530	0.313
DP2	3.78	16.86	40.10	31.04	44.00	− 0.003	− 0.505	0.863
DP3	4.30	8.00	49.20	42.17	47.78	− 0.616	0.681	0.397

Best factor was chosen based on factor that has the most possible dependent variable weight that related to the disease interest from RRR analysis. DP1: high SFA, high DED, high sodium, DP2: low SFA, low DED, high sodium, DP3: low SFA, high DED, high sodium. *DED* Dietary energy density, *SFA* Saturated fatty acid.

A higher proportion of married participants, with high stress score, and elevated triglyceride (TG) level were found in higher tertiles of DP1. In contrast, participants at higher tertiles of DP1 have considerably lower mean BMI compared to lower tertiles (Tables 1, 2). Intake of DED, fat, SFA, sodium, and main food groups such as seasoning sauce, salted and processed foods, coconut-based dishes, fast foods and snacks, rice dishes, and fat spread were shown to be the highest in tertile 3, reflecting the features of identified DPs. On the other hand, participants with higher adherence to DP1 had significantly lower intake of carbohydrate, protein, fibre, and other food groups (fruits, green leafy vegetables, white rice and other vegetables) compared to their counterparts with lower adherence to this DP (Table 4).

**Table 4:** Dietary intake of participants in main dietary patterns based on tertiles (N = 365).

Dietary intake	Total (N = 365)	Dietary pattern 1 (High DED, high SFA, high sodium)			
		Tertile 1 (N = 121)	Tertile 2 (N = 122)	Tertile 3 (N = 122)	*p* value*
**Nutrient intake**					
Energy intake(kcal/day)	1815 ± 629	1715.7 ± 641	1843.9 ± 625.8	1884.7 ± 612.6	0.092^b^
Energy density (kcal/g)	1.58 ± 0.3	1.38 ± 0.3	1.6 ± 0.2	1.7 ± 0.2	< 0.001^b^
Carbohydrate (%EI)	56.1 ± 8.3	61.7 ± 7.5	56.2 ± 5.6	50.3 ± 7.4	< 0.001^b^
Protein (%EI)	16.8 ± 3.5	16.0 ± 3.8	16.8 ± 3.3	17.6 ± 3.3	< 0.002^b^
Fat (%EI)	27.2 ± 6.1	22.2 ± 5.1	27.0 ± 3.3	32.2 ± 5.2	< 0.001^b^
Saturated fatty acids (g/1000 kcal)	13.3 ± 3.4	10.2 ± 2.2	13.2 ± 1.5	16.4 ± 2.8	< 0.001^b^
Sodium (mg/1000 kcal)	1503.2 ± 592.0	1356.2 ± 469.8	1424.2 ± 438.5	1728.0 ± 751.1	< 0.001^b^
Fiber density (g/1000 kcal)	5.4 ± 3.0	6.6 ± 3.8	4.8 ± 1.9	4.7 ± 2.5	< 0.001^b^
**Main food groups (g/day)**					
Seasoning sauce	15.0 ± 18.1	9.9 ± 11.1	13.2 ± 12.0	21.8 ± 25.3.4	< 0.001^b^
Salted and processed foods	17.5 ± 20.6	13.5 ± 16.3	16.2 ± 17.7	22.6 ± 25.4	0.002^b^
Coconut based dishes	50.9 ± 62.8	33.2 ± 41.3	56.3 ± 73.8	63.0 ± 65.0	< 0.001^b^
Fast foods and snacks	15.1 ± 23.5	10.5 ± 17.2	13.7 ± 20.1	21.2 ± 30.0	0.001^b^
Rice dishes	99.2 ± 83.3	75.0 ± 72.1	108.1 ± 79.6	114.3 ± 92.0	< 0.001^b^
Fat spread	2.3 ± 5.7	1.2 ± 3.8	2.3 ± 6.2	3.4 ± 6.6	0.009^b^
Fruits	137.5 ± 201.4	221.6 ± 300.4	98.7 ± 110.5	93.0 ± 98.7	< 0.001^b^
Green leafy vegetables	70.9 ± 79.4	102.3 ± 107.1	58.8 ± 59.7	51.9 ± 50.0	< 0.001^b^
White rice	225.5 ± 125.0	254.8 ± 138.5	248.6 ± 128.7	173.5 ± 85.3	< 0.001^b^
Other vegetables (e.g., pod and seed, root, marrow vegetables)	54.1 ± 72.4	77.9 ± 102.3	43.2 ± 48.8	41.6 ± 46.4	< 0.001^b^

^b^One-Way ANOVA. *Significant level at *p* value < 0.05. *DED* Dietary energy density, *SFA* Saturated fatty acid.

### Association between DP ‘High DED, high SFA, high sodium’ and CHD

Table 5 shows the unadjusted and adjusted associations between DP1 z-scores and the risk of CHD for the total study population. Logistic regression analysis showed that there was a significant association between DP1 (z-score) and CHD in the adjusted models. For DP1, with an increase of 1 SD unit of DP1 z-score, there was increase odds of CHD by 32% (OR = 1.32; 95% CI: 1.03, 1.69) *p* value = 0.026, after adjusting for age, sex, race, education level, income, family history of CHD, marital status, smoking, physical activity, stress level and BMI. However, no significant association was reported between the DP1 and CHD in this study population based on adherence levels throughout the DP’s tertiles (Table 6).

**Table 5:** Association between dietary pattern ‘High SFA, high DED, high sodium’ and coronary heart disease (CHD).

Model	OR	95% CI	*p* value
Unadjusted	1.29	1.06, 1.56	0.010
Model 1^a^	1.29	1.05, 1.57	0.013
Model 2^b^	1.29	1.02, 1.63	0.036
Model 3^c^	1.32	1.03, 1.69	0.026

^a^Model 1: adjusted for age and sex.

^b^Model 2: age, sex, race, education level, income, family history, marital status.

^c^Model 3: age, sex, race, education level, income, family history of CHD, marital status, smoking, physical activity, stress level, BMI. Multicollinearity was checked using variance inflation factor (VIF) for each independent variable in the model (VIF < 5: no correlation found). Binary logistic regression was used to test the association of DP1 with CHD *Significant level at *p* value < 0.05.

*CI* Confidence interval, *OR* Odd ratio.

**Table 6:** Association between the tertiles of dietary pattern ‘High SFA, high DED, high sodium’ and coronary heart disease (CHD).

Model	Tertiles of DP ‘High SFA, high DED, high sodium’					
	2nd tertile vs. 1st tertile			3rd tertile vs. 1st tertile		
	OR	95% CI	*p* value	OR	95% CI	*p* value
Unadjusted	0.81	0.49, 1.34	0.406	1.24	0.75, 2.05	0.405
Model 1^a^	0.74	0.44, 1.26	0.265	1.17	0.69,1.97	0.564
Model 2^b^	0.93	0.51, 1.70	0.821	1.18	0.65,2.15	0.583
Model 3^c^	0.95	0.51, 1.77	0.872	1.30	0.70,2.43	0.413

^a^Model 1: adjusted for age and sex.

^b^Model 2: age, sex, race, education level, income, family history, marital status.

^c^Model 3: age, sex, race, education level, income, family history of CHD, marital status, smoking, physical activity, stress level and BMI. Multicollinearity was checked using variance inflation factor (VIF) for each independent variable in the model (VIF < 5: no correlation found). Binary logistic regression was used to test the association of tertiles of DP1 with CHD *Significant level at *p* value < 0.05. *CI* Confidence interval, *DED* Dietary energy density, *OR*: odd ratio.

## Discussion

In Malaysia, the role of DPs on CHD risk is still unclear. This study was conducted to provide a better insight into the link between DPs and CHD among high risk adults with MetS in Malaysia. We found a significant association between DP1 (z-scores) with CHD which was independent of age, sex, race, education level, income, family history, marital status, smoking, physical activity, stress level and BMI. However, no significant association reported when compared across the DPs tertiles.

The main DP in this study was characterized by high consumption of coconut-based dishes, fast foods and snacks, rice dishes, fat spread, seasoning sauces, salted and processed foods; and low intake for fruits, green leafy vegetables, white rice and other vegetables. This DP is align with the previous studies that found a link between a “Western” DP with CHD, as well as the benefits of adherence to Dietary Approach to Stop Hypertension (DASH-style) diet to reduce the risk of CHD or CVDs^40,41^. The features of this DP has a little resemblance with a DP identified in the Whitehall II study from the UK, which found that high intakes of white bread, fried potatoes, sugar in tea and coffee, burgers and sausages, soft drinks, and with low consumption of French dressing, and vegetable, and it has become a predictive DP of CHD incidence among men and women aged 35–55 years old after 15 years of follow up^42^. The resemblance was particularly related to the high intakes of fast foods, processed foods and low intake of vegetables.

The high sodium pattern in the identified DP in this study was predominantly contributed by a high intake of seasoning sauce, salted and processed foods. The strong link between CHD for DP1 that was featured by high sodium intake as one of its response variables in this study, is consistent with another study which showed the association between a “high-salt” DP with a higher 10-year ischemic CVD risk score level among Chinese coal miners^43^. A previous meta-analysis also had an unequivocal agreement that high salt intake was associated with a significantly higher risk of total CVD^44^. The salt-rich diet in this study might be slightly different from the salt-rich diet in previous Westernized studies due to food culture and availability, also an eating pattern divergence. Apart from the processed and manufactured foods, sodium-rich foods in the high salt diet from this study were predominantly from seasoning, condiments, sauces and locally produced high-salt foods which are consumed by the population from various ethnicities in Malaysia including the Malay, Chinese and Indian. The most common locally produced high-salt foods among Malaysian population include salted fish, salted egg, shrimp paste, anchovy sauce, salted vegetables, fish crackers etc. are the addition to the common processed or manufactured foods such as instant noodles, sausages, snack and canned food. As expected, this study observed that daily sodium intake and systolic blood pressure were significantly higher among CHD participants compared to the non-CHD participants. The link between high systolic blood pressure (> 115 mmHg) and increased risk of CHD and other CVDs was consistent with the previous studies^45,46^. The high sodium intake and the increase in blood pressure levels are related to water retention, increase in systemic peripheral resistance, alterations in the endothelial function, and changes in the structure and function of large elastic arteries, by which elevated blood pressure particularly over a long period of time, puts and incredible strain on the heart^47^. While WHO has recommended reducing salt intake to less than 5 g (sodium < 2000 mg/day) per day for adults^48^, the CHD group in this study had consumed nearly 3100 mg/day of sodium which was 55% exceeded the daily recommended intake for sodium.

Fat spread, coconut-based dishes, fast foods and snacks were the main high calorie and high fat foods found in DP1. Several features of high DED and high SFA pattern in this study were comparable to other DPs from previous studies which reported that DP high in hydrogenated fat and Western DP were significantly associated with higher blood lipids and greater risk of CHD events^49,50^. High SFA foods in DP1, mainly contributed from coconut-based dishes, fast foods and snacks, rice dishes and fat spread, were strongly linked with the likelihood of having CHD and this finding was in line with the previous studies showing that SFA was the dominant dietary culprit of CVDs through elevation of plasma LDL cholesterol level^51,52^. Previous studies also reported that reductions in dietary fat and SFA reduced CHD risk and cardiovascular events^34^. Several evidence have reported the link between even-chained saturated fatty acids (e.g., palmitic acid (16:0), stearic acid (18:0)), which is abundant in animal fats as well as palm oil products with CVDs and CHD risk^53,54^. Even-chained SFA rich ingredients (e.g., butter, cheese, laminating fat, shortening, margarine, palm oil), are widely used in the food industry and were consumed in large quantities in this DP, primarily from fast foods and snacks, rice dishes, and fat spread.

Generally, SFA is the main fatty acids in coconut milk and coconut flesh^55^, which are the common main ingredients in Malaysian cuisines. The use of coconut milk is mainly found in Malaysian local coconut-based entrees and rice dishes, and these food groups were included in DP1. Although the effects of coconut milk with CHD is quite controversial, this study has shown a positive association between DP containing coconut-based foods with CHD. It is not viable, however, to conclude that high coconut milk intake will lead to CHD as DP1 in this study was based on the combination of several other foods groups including fast foods, snacks, and fat spread. Nonetheless, a previous study reported that frequent consumptions of coconut milk of > 3 times per week increased risk of vascular disease^56^. In contrast, an earlier study found that SFA intake, including coconut, was not a predictor of CHD among coconut-consuming Minangkabau in West Sumatra Indonesia^57^. Furthermore, an ecological study on health effect of coconut among Sri Lanka population, concluded that the rise in cardiac morbidity and mortality over the years was unlikely to be due to the consumption of coconuts; while other factors other than coconut consumption may be the predictors of cardiovascular mortality^58^. Direct causal relationship between coconut fat and CVDs or CHD risk, is inconclusive as several studies even showed beneficial effect of coconut milk and coconut oil on lipid profile and CVD risk^59,60^. Thus, even though coconut contains primarily SFA, it is fundamental for the researcher to look at the length of SFA chain too, since different chain of SFA act differently metabolically and have different health effects^61^. These rather contradicting findings have led to inconclusive evidence, therefore further prospective longitudinal studies on coconut based foods, impacts of different types of SFA on health outcomes and CHD risk may be required to confim this association.

As anticipated, this study found that, low intake of fruits, green leafy vegetables, and other vegetables (pod and seed, root, marrow vegetables), which is the negative loadings of DP1, was associated with CHD. Adequate vegetable and fruit consumptions are always the cornerstones of a healthy diet. A recent meta-analysis has proven that a higher total fruit and vegetables intake was associated with decreased CHD incidence^62^. Several other studies also found a link between increased intake of leafy green vegetables and lower CHD risk, as high vitamin K and nitrates in these foods can improve arterial function^63^. Although, the DP in this study portrayed the pattern of low intake of vegetables and fruits, it did not highlight the effect of main soluble fibre foods particularly from the whole grains. Many previous studies have shed the light on the association between whole grain-rich foods with CHD^64^, however, this study was unable to provide conclusive evidence about it.

To our knowledge, this is the first study to identify a DP associated with CHD among adults with MetS in Malaysia using RRR. The RRR approach is a holistic method that combines both exploratory approach and hypothesis-oriented approach and it presents the results based on disease outcomes of interest. Dietary patterns are largely dependent on the population’s food culture and environmental factors, and they vary between populations. This information is practically translatable and useful in assisting local health professional in providing useful food-based information to the public in addition to nutrient recommendations which are tailored to the Malaysian population, to achieve meaningful dietary changes.

Among the limitations of the present study, it’s important to note that, the cross-sectional design of this study was unable to confirm the causality of the disease, with the potential of the reverse causality which may explains some variability in the results. There was no adjustment done for measurement error (e.g., anthropometry (WC), biochemistry, and clinical data), since these variables involve in the causal pathway between exposure and outcome. Since biological parameters were obtained from medical records, some different kits with different precision might be used. There was a possibility of bias in relation to dietary reporting by the study participants using the FFQ which was relied on the participant’s memories and the FFQ are prone to systematic error. However, we tried to minimise this bias, by conducting researchers-led interviews for the FFQ instead of self-reported. In addition, any extreme values of dietary intake was dealt prior to analysis, in which unreliable dietary energy intake below than 500 kcal/day for men and women and exceeding 5000 kcal/day for women or 8000 kcal/day for men was excluded for DP analysis^65^.

In the future studies, we firmly believed that a larger sample size with more restrictive selection criteria is required to confirm the CHD risk factors in Malaysia, thus can decrease the possibility of selection bias. A larger sample size was hardly attained in this study due to several challenges, most notably, time and financial constraints, logistical problems that required researchers to collect samples from all regions of Malaysia within a limited time frame, and participant recruitment difficulties especially during pandemic COVID-19. Refusal of subjects to participate in the exposure and disease-related study, or so called as self-selection bias, had led to this unintended effect. Nonetheless, the findings of this study are representative from all five main regions of Malaysia. More robust longitudinal prospective studies are required to confirm this association.

## Conclusion

The main DP found in this study was DP1 “High SFA, high DED, high sodium” has shown a significant association with CHD after adjustment of other covariates, but no association was found when compared across the DPs tertiles. The DP reflects the real dietary patterns that represent the food intake of the high-risk of CHD adults with MetS in Malaysia.

## Supplementary Information

Supplementary Information.

## Data Availability

The datasets generated and analysed during the current study are not publicly available due to sensitivity of personal data but are available from the corresponding author on reasonable request.
