# Supplementary material for: Association between a dietary pattern high in saturated fatty acids, dietary energy density, and sodium with coronary heart disease
Source: Sci Rep. 2022 Jul 29;12:13049. doi: 10.1038/s41598-022-17388-5 (PMC9336144; doi:10.1038/s41598-022-17388-5)
Supplement: Supplementary file 1 — Supplementary Information. [file 41598_2022_17388_MOESM1_ESM.pdf]

Association between a dietary pattern high in saturated fatty acids, dietary energy density, and sodium with coronary heart disease

Nur Ain Fatinah Abu Bakar[1], Aryati Ahmad[1,2*], Wan Zulaika Wan Musa[1&] Mohd Razif Shahril[3], Nadiah Wan-Arfah[1], Hazreen Abdul Majid[4], Carmen Piernas[2], Ahmad Wazi Ramli[5], Nyi Nyi Naing[6]

Nutrients	Univariate analysis OR (95% CI)	p value
**Energy intake (kcal/day)**	1.001(1.001,1.002)	<0.001
**Dietary energy density (DED) kcal/g**	1.26(0.60, 2.65)	0.550
**Fat % of EI**	1.07(1.03, 1.11)	<0.001
**SFA (g/day)**	1.09(1.06, 1.11)	<0.001
**SFA (g/1000 kcal)**	1.10(1.04, 1.18)	0.003
**Sodium (g/day)**	1.00(1.00, 1.001)	<0.001
**Sodium (mg/1000kcal)**	1.00(1.00, 1.00)	0.553

Table S1. Simple logistic regression between single nutrients and CHD (n=365).

Binary logistic regression was used to test the association of each nutrient with CHD *Significant level at p-value <0.05. SFA: Saturated fatty acid. Abbreviations: CI: Confidence interval; DED: Dietary energy density; SFA: saturated fatty acids.
